# Micro and Nano Interdigitated Electrode Array (IDEA)-Based MEMS/NEMS as Electrochemical Transducers: A Review

**DOI:** 10.3390/nano12234171

**Published:** 2022-11-24

**Authors:** Elyana Kosri, Fatimah Ibrahim, Aung Thiha, Marc Madou

**Affiliations:** 1 Department of Biomedical Engineering, Faculty of Engineering, Universiti Malaya, Kuala Lumpur 50603, Malaysia; 2 Centre for Innovation in Medical Engineering (CIME), Faculty of Engineering, Universiti Malaya, Kuala Lumpur 50603, Malaysia; 3 Centre of Printable Electronics, Universiti Malaya, Kuala Lumpur 50603, Malaysia; 4 Department of Mechanical and Aerospace Engineering, University of California Irvine, Irvine, CA 92697, USA; 5 School of Engineering and Sciences, Tecnologico de Monterrey, Monterrey 64849, NL, Mexico; 6 Academia Mexicana de Ciencias, Ciudad de México 14400, CDMX, Mexico

**Keywords:** interdigitated electrode array, carbon MEMS, cyclic voltammetry, electrochemical analysis, nanocomposites, nanoparticles, electrochemical transducer, biosensor

## Abstract

Micro and nano interdigitated electrode array (µ/n-IDEA) configurations are prominent working electrodes in the fabrication of electrochemical sensors/biosensors, as their design benefits sensor achievement. This paper reviews µ/n-IDEA as working electrodes in four-electrode electrochemical sensors in terms of two-dimensional (2D) planar IDEA and three-dimensional (3D) IDEA configurations using carbon or metal as the starting materials. In this regard, the enhancement of IDEAs-based biosensors focuses on controlling the width and gap measurements between the adjacent fingers and increases the IDEA’s height. Several distinctive methods used to expand the surface area of 3D IDEAs, such as a unique 3D IDEA design, integration of mesh, microchannel, vertically aligned carbon nanotubes (VACNT), and nanoparticles, are demonstrated and discussed. More notably, the conventional four-electrode system, consisting of reference and counter electrodes will be compared to the highly novel two-electrode system that adopts IDEA’s shape. Compared to the 2D planar IDEA, the expansion of the surface area in 3D IDEAs demonstrated significant changes in the performance of electrochemical sensors. Furthermore, the challenges faced by current IDEAs-based electrochemical biosensors and their potential solutions for future directions are presented herein.

## 1. Introduction

**Figure 1:**
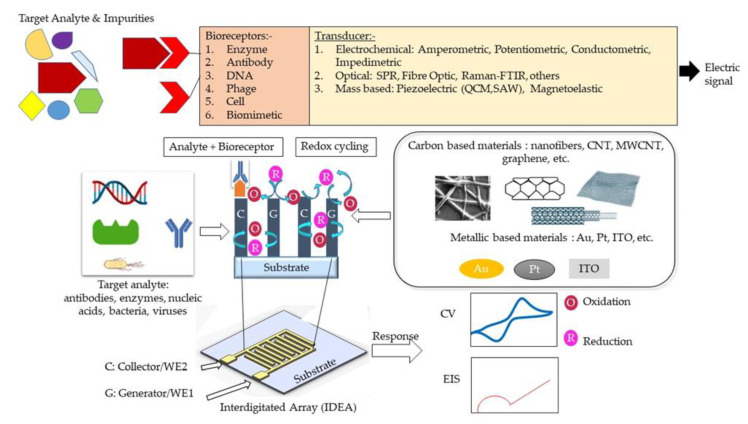
Schematic design of IDEA design-based electrochemical detection (amperometric and impedimetric) methods.

A biosensor typically consists of three main elements, namely a bioreceptor, transducer, and signal processing system [1]. A bioreceptor, also known as biological recognition element, involves an immobilized biocomponent, which is capable of detecting a specific target analyte [2]. Nucleic acid, enzymes, antibodies, cells, etc., are types of biocomponents. A transducer is a converter that converts a biochemical signal into an electrical signal [3]. The reaction between a bioreceptor and target analytes generates distinct chemical reactions, such as electron flow, release of heat, and changes in pH or mass, subsequently creating new chemicals. The detection of an electrical signal by the transducer is amplified and sent to microelectronics and data processors for signal measurement in terms of a print out, an optical change, or as digital display. Biosensors can be categorized into bioreceptors and transducers. Bioreceptors consist of biomimetics, enzymes, phages, DNA, cells, and antibodies. The transducer can be further divided into three categories, which are: (i) those based on electrochemical transducers, such as electrical impedance spectroscopy (EIS), potentiometric, amperometric, and conductometric; (ii) mass-based, such as piezoelectric and magnetoelastic; and (iii) optical-based biosensors, such as chemiluminescence, fluorescence, surface plasmon resonance (SPR), fibre optic, and others. The classification of biosensors has been described in several pieces of literature and is illustrated in Figure 1 [4,5,6]. 

The electrochemical biosensing techniques can be divided into amperometric, impedimetric, conductometric, and potentiometric sensing [5]. In an amperometric sensor, one measures the current response at a fixed potential to detect the concentration of an analyte [7]. In a potentiometric sensor, potential changes at a working/sensing electrode are measured with respect to a reference electrode and under the conditions of constant current (i.e., typically zero). Conductometric sensors measure the electrolytic conductivity to monitor the progress of a reaction. An impedimetric sensor works by measuring an impedance change while applying a small sinusoidal voltage that is varied over a range of frequencies. Electrochemical sensors are among the most popular biosensors due to their simplicity, good-to-excellent limit of detection (LOD), high selectivity, and ease of fabrication, as well as the promising opportunity for miniaturization and low-cost fabrication [6,8,9,10]. Innovative electrodes in electrochemical cells can be mass-manufactured using a variety of materials and economical manufacturing processes [11,12,13]. Moreover, integrated circuit technologies make it possible to integrate electrodes with electronics for further biosensor miniaturization [14,15].

A basic electrochemical three-electrode cell consists of a working electrode, a counter electrode, and a reference electrode. The working electrode is the actual transduction element for the electrochemical reaction at the electrode/analyte solution interface. A current at the working electrode is offset by an equal but opposite current at the counter electrode, and hence there is no current flow between the working and high-input impedance reference electrode, allowing us to accurately track changes in the working electrode potential. The counter electrode must be of large surface area (since the current through the cell must be controlled by the reaction at the working electrode) with a stable and good conductor [16]. Carbon [17], platinum (Pt) [18], gold (Au) [19], and other materials [7,20,21] are the common materials used to fabricate the counter electrode. Reference electrodes are designed so that an equilibrium is set up with a known potential between a metal wire and the surrounding solution. A silver/silver chloride (Ag/AgCl) system is widely used as a reference electrode [22,23].

Despite the booming interests in IDEAs that started more than 30 years ago, current studies manipulate materials from different sources to suit the electrode usage of IDEA. Interdigitated electrode array (IDEA) from carbon and metal sources, in contrast, emerges as a favorable electrode biosensor for various health-monitoring and biomedical applications. Indeed, numerous pieces of research highlighting the development of an IDEAs-based biosensor with various detection methods have been extensively reported [24,25,26]. Enhanced signal amplification allowing detection of low-concentration bioanalytes displayed by an IDEAs-based biosensor proved to be advantageous for electrochemical biosensing [27,28,29]. With an IDEA configuration, the limit of detection (LOD) of a biosensor can be significantly improved [30]. In such a configuration, two comb-shaped working electrodes are arranged in an interdigitated manner, as presented in Figure 1 [31,32]. IDEAs are widely used as impedimetric transducers in electrochemical impedance spectroscopy (EIS) [33,34,35]. EIS employs a controlled alternating current (AC) with electrical stimulus between 5 and 10 mV to measure small variations in capacitance/resistance caused by analyte and electrode surface interactions. These capacitance and resistance changes are due to changes in faradaic (electron transfer/resistance changes) and non-faradaic (dielectric/capacitance changes) processes at the electrode surface [36]. The signal strength of an IDEA-based biosensor can be controlled through the optimization of the active area, width, and spacing of the electrode fingers [37,38,39,40]. Hence, they can be used for real-time, label-free, and in situ detection of target analytes [41]. However, the main drawback of these EIS-based sensors is their poor detection limit, compared to other electrochemical methods [5]. Contrastingly, a combination of electrochemical sensing techniques, for example, amperometry and impedimetry could enhance biosensor performance [42]. The details on different electrochemical detection methods are presented in Table 1.

When IDEAs are used in an amperometric redox amplifying biosensors, the interdigitated combs/fingers are called generator and collector electrodes. Redox cycling [43,44] occurs when redox species generated at the generator electrode (in an oxidation reaction) are collected at the collector electrode (in a reduction reaction) [45]. For this to occur, the generator and collector electrodes must be spaced close enough to overlap the diffusion layers of the redox species between the two electrodes [30,46]. A small gap between the finger electrodes allows for reversible redox species to undergo repeated oxidations/reductions (redox-cycling) before diffusing out to the bulk solution. The redox amplification factor is the ratio of the generator current in dual-mode operation (i.e., the generator and collector are at different enough potentials to allow for redox amplification) to the generator current in single mode (the generator and collector are at the same potential). The collection efficiency [47,48] is the ratio of the collector current to the generator current [49,50] or the ratio of the cathodic current to the anodic current at steady state [51]. The smaller the gap between adjacent comb electrodes, the shorter the diffusion time for the redox species to diffuse across the gap, resulting in a higher current amplification factor [46,49,52]. Due to redox amplification, IDEA-based electrochemical biosensors exhibit high signal-to-noise ratios, and thus better LODs, low ohmic drops, and rapid response time [53]. Furthermore, the efficiency of redox cycling and redox amplification factors can be further improved by increasing the height of the two working electrodes in a three-dimensional form [54]. The three-dimensional IDEAs (3D IDEAs) with their higher aspect ratio improve the contribution of linear diffusion between the electrode sidewalls and increase the IDEA’s electrode surface overall area [49]. Another favorable microfabrication strategy, called the carbon microelectromechanical systems (C-MEMS) [55], offers the fabrication of high aspect ratio carbon IDEAs and is rendered simple and inexpensive through a one-step photolithography step of a polymer carbon precursor and subsequent pyrolysis [54,56].

The IDEA-based electrochemical biosensors provide easy-to-use and cost-effective fabrication, making them good candidates for portable point-of-care (POC) diagnostic devices [42,57]. This review focuses on the micro and nano size of IDEA-based electrochemical biosensors, highlighting preferential fabrication techniques, including C-MEMS/NEMS technique, and their contributing factors to improve the sensitivity and selectivity of the electrochemical sensors by utilizing the amperometric and electrical impedance spectroscopy (EIS) detections for biomedical applications. This paper presents and discusses four-electrode configurations in electrochemical sensors using IDEAs as working electrodes in biomedical applications. Furthermore, a comparison between 2D IDEAs and 3D IDEAs was presented, focusing on the IDEA’s width, gap, height modification, methods of increasing the 3D IDEA’s surface area, and integration of nanoparticles to improve the sensitivity of the sensor performance.

**Table 1:** The working principle of electrochemical biosensors and their advantages and disadvantages.

Type of Electrochemical Biosensor	Working Principle	Advantages	Disadvantages	Refs.
Amperometric	Measures current resulting from redox cycling at a constant voltage (CA) and controlled potential (CV).	- Low fabrication cost; - High sensitivity.	- A signal reduction from fouling agents, and interferents in a sample.	[58,59]
Impedimetric	Measures impedance and changes in ionic concentration under no current flow between reference and ion-selective electrodes.	- Detect current changes without redox reaction; - Simple detection method.	- Slow dynamic response. - Low detection method;	[5,60]
Conductometric	Measures of conductance and interfacial electric arise from the biorecognition process.	- High signal-to-noise (S/N) ratio; - Directly detect the binding events; - No interference.	- Slow response; - Accuracy of detection depends on instrumental and experiment procedures.	[61]
Potentiometric	Measures potential difference from changes in ion concentration.	- No reference electrode; - Efficient at low amplitude alternating voltage; - Simplicity.	- Low specificity; - Low S/N ratio.	[62,63]

CV = cyclic voltammetry, CA = chronoamperometry.

## 2. Features in IDEA

### 2.1. Electrical Double Layer (EDL)

**Figure 2:**
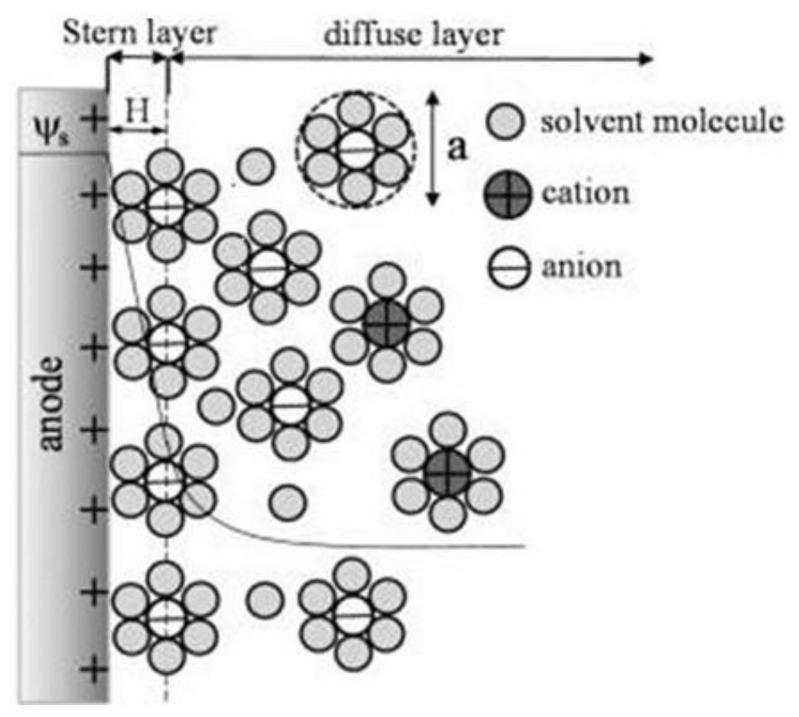
Schematic of the electric double layer structure showing the arrangement of solvated anions and cations near the electrode/electrolyte interface in the Stern layer and the diffuse layer of Gouy–Chapman–Stern model. Reprinted with permission from Ref. [75]. Copyright © 2022, American Chemical Society.

Figure 1 shows how the electrochemical IDEA-based sensor requires the interaction between target analyte/bioreceptor/electrolytic solutions and the sensor surface to produce an electrical signal. However, due to the various material sources for producing the electrode, such as carbon and metal, the interactions between the electrolytic solutions with the sensor create a phenomenon called electrical double layer (EDL) on the electrode surface [64,65,66,67,68]. The EDL is formed when the electrons in the electrode surface (e.g., metal) interact with ions in the electrolyte solution. Referring to the Gouy–Chapman–Stern (GCS) EDL model, the liquid solution creates two layers consisting of a compact layer and a diffuse layer [69]. The compact layer consists of the immobile solvent ions and molecules that adsorb into the solution/material interface, whereas the diffuse layer contains the mobile solution that carries solvated electroactive and inactive ions or the net charge within the liquid solution (Figure 2). The scattering of charges in the diffuse layer is determined by the Debye length, thus, providing the surface potential or charge of the material [70]. The EDL affected the electrochemical performance.

Yang et al. utilized computer simulation to study the effect of EDL in nanometer single electrode structure via the voltammetric performance. They reported that the extension of the diffuse layer into the diffusion layer in the EDL caused the increasing charge valence or the absence of the supporting electrolyte in the solution, which affected the current response of the nanometer electrode. It was reported that modifications to the thickness of the EDL compact layer and its relative permittivity significantly influenced the current response [68,69,71]. Moreover, the ultramicroelectrodes, ranging from 25 µm to the submicrometer employed in published electrochemical experiments, were observed to cause nonlinear diffusion effects, resulting in enhanced mass transport, higher steady-state redox reaction rates, and faster response time, compared to larger electrodes [50,72,73,74].

### 2.2. Crucial Parameters of the Sensor

Generally, the important parameters in performing biosensing are determined by sensitivity, selectivity, specificity, and limit of detection (LOD). In particular, the labelling method for target molecules is necessary for specificity, whereas the label-free method is more common for electrochemical detection using redox reaction [76]. The sensitivity of the electrochemical measurement method depends on the relations among the electron transfer between the molecule and the electrode, creating significant amounts of electrical current without any label. 

In IDEA, the width and gap between adjacent fingers are crucial in the fabrication of IDEA-based electrochemical sensors. A smaller width will result in smaller capacitance, and hence faster mass transport of the species. In the pursuit to enhance the sensitivity of IDEA-based biosensors with lower detection limits, the design of IDEA itself offers practical manipulation in terms of increasing the number of widths within the array or lengthening the width. As a result, simple modifications may lead to higher faradaic current to capacitive current ratios and higher signal-to-noise ratios, producing highly sensitive biosensors suitable for biosensing applications. Furthermore, reducing the gap between adjacent fingers leads to a higher diffusional flux of redox species and enhances the rapid response at the collector, as well as sensor sensitivity [77]. 

Another factor to improve sensitivity of the biosensors for amperometric detection is redox cycling. Redox cycling between the narrow gap of the generator and collector of IDEA increase current response and signal-to-noise ratio [69,78]. A notable example was shown through work by Huang et al. [79] in which smaller gaps between electrodes, such as a 300 nm gap, were required for the dopamine detection of 2.89 nA/µM to achieve high feedback between the generator and collector electrode. The smaller gap size between adjacent fingers may enhance the current amplification due to the shorter diffusion time between the two electrodes’ fingers [46,52,80]. 

Redox amplification also improves the selectivity of the biosensor, as it can selectively amplify reversible redox species of interest over irreversible species [30,49]. The specificity of the biosensor can be determined by the specificity of the biologically active materials and the target analytes. In this case, a probe incorporated with targeted biological components and a transducer convert the biochemical signals into electrochemical, acoustic, etc., [81]. In comparison, the optical method requires fluorescence detection or chemiluminescence with labels for biosensing detection. For example, green fluorescent protein (GFP) is a powerful tool to genetically encode a protein of interest (POI) for protein-based detection.

## 3. Micro and Nano IDEA

IDEA-based electrochemical sensors offer much potential in terms of the exploration of their functionality for practical biosensing applications. Considerable attention has been provided to unravelling the best substrates, electrode materials, and fabrication techniques and their influence on the IDEA’s structures and geometries (e.g., width, the gap between adjacent fingers, and height), as well as sensitivity outcome [82,83,84]. Dizon et al. reported that the micron unit eased the analysis with a small sample size and helped to increase the sensor sensitivity for EIS measurement via the micron unit separation between working and counter electrodes [29]. Research found that IDEA is one of the versatile electrodes that allows the expansion of a three-electrode configuration (one working electrode) to a four-electrode configuration (two working electrodes) system. This is due to the advantages of the pair of comb fingers with the reference and counter electrodes available in the sensor setup. Moreover, miniaturized IDEA-based electrochemical sensors rose to fame, as reported in many recent studies, due to retainable electrode width, gap, and height in micro and nano units [85,86,87]. 

Interestingly, a preliminary study on the electric double layer (EDL), electric field distribution, and current density of IDEA can be performed numerically using finite element analysis (FEA) [88] software, such as COMSOL Multiphysics and ELECTRO. Finite element analysis simulation gives an early insight into the experimental outcomes and minimizes design errors [89,90,91,92]. The digital simulation helps researchers to predict the IDEA’s electrical performance by virtually varying the height, gap, and width of IDEA [46,48,69,93,94,95]. The experimental results may differ from the simulation results as fabricated electrode dimensions and resultant EDL [69] may vary, affecting the overall sensor measurement [91,96]. Despite that, computational simulation offers early analysis of the sensor sensitivity from various electrode geometries and configurations, prior to actual fabrication.

### 3.1. Substrate, Electrode Material, and Fabrication Techniques

The substrates, electrode materials, and fabrication techniques are important criteria when fabricating IDEA-based electrochemical sensors. Most notable pieces of research employed carbon and metals as the source material of electrodes for IDEA by leveraging their advantageous properties, such as the semiconducting nature and excellent thermal and chemical properties that produce functional sensors with excellent sensitivity and selectivity.

#### 3.1.1. Metal-Based IDEA

In addition to carbon-based IDEAs, metal/noble metals, such as Au, Pt, Al, Fe, and organic/inorganic-based nanoparticles, have been used as IDEA’s electrode materials, with gold (Au) [91] or platinum (Pt) being the most popular selections for IDEA’s starting materials [97]. Gold has many favorable properties, such as inertness, biocompatibility, resistance to oxidation, high conductivity, compatibility with surface functionalization methods, and suitability to being manufactured in ranges of nanosizes [98,99]. The advantages of gold have been employed in the development of highly sensitive, selective, and stable analytical devices in many biosensing applications [100,101]. Thus, several reports on IDEA-based metallic sources are presented for comparison in terms of fabrication methods and sensor achievements.

The important types of substrates and fabrication techniques have been highlighted and compared. Rishi et al. studied three types of IDEA fabrication techniques and substrates (copper-cladded IDEA, laser-induced graphene IDEA from polyimide sheet (LIG-based IDEA), and 3D-printed graphene filament IDEA) with constant width = 917 µm, gap = 553 µm, and all three IDEA sensors tested for *Escherichia coli* (*E. coli*)-sensing using impedimetric sensing [102]. In this research, they found that the LIG-based IDEA presented the best sensitivity, with detection (LOD) as low as 2.5 CFU/mL, tested using *E. coli*, and the best selectivity by producing the least amount of interference in the presence of 1.008 × 10^5^ CFU/mL *E. coli* and Shewanella Oneidensis bacteria concentration, respectively. Moreover, the LIG technique was reported to have a relatively short fabrication time with the cheapest costs, compared to the other two techniques. 

Another commonly used material to fabricate IDEA is platinum (Pt). For instance, Matylitskaya et al. [18] fabricated Pt nanogap IDEAs (nIDEAs) on silicon (Si) substrate for Lab-on-a-Chip applications (LoC). Ferrocenemethanol (FcMeOH) and p-aminophenol (pAP) were used as the redox couples to perform CV and CA electrochemical characterization of Pt nIDEAs. The Pt nIDEAs (g = w = h = 100 nm) obtained an amplification factor of 161 with 1 mM FcMeOH and an amplification factor of 118 with 1 mM pAP, respectively, with collection efficiency of more than 99% for both tested FcMeOH and pAP. Thus, Pt can be considered as one of the promising metallic materials in fabricating IDEA-based biosensors with enhanced performance and sensitivity [103,104]. 

Taking advantage of gold (Au) as IDEA’s electrode material, a combination of two Au-IDEAs in supercapacitors has been studied by Ferreira et al. [105]. They created a supercapacitor using an electropolymerization method to graft the polypyrrole/carbon nanotube (PPy/CNT) nanohybrid film on two Au-IDEAs (∼60 nm thickness, 10 μm width, and 10 μm gap) followed by investigation on electrochemical analysis, impedimetric sensing, and PPy/CNT nanocomposite synthesis. They immobilized Anti-Cystatin C (Anti-CysC) on IDEA via ethylenediamine bifunctional agent, glycine blocking in acid, and alkaline medium using covalent entrapment. Based on their result, the IDEA immunosensor to CysC capacitive effect of the antigen–antibody interaction serum was detected by double-layer capacitance under low frequency and the response was measured by changes on the phase angle with a linear range of up to 300 ng/mL. They also calculated the cut-off point for the serum sample and their results showed a total reduction in non-specific binding at approximately 28 ng/mL CysC. To maximally minimize non-specific binding, the blocking agent of glycine was used in two different mediums (alkaline and acid) for the IDEA immunosensor [105]. Oh et al. studied the label-free electrochemical immunosensor using gold IDEA (Au-IDEA) modified with self-assembled monolayers (SAMs) and IL-6 antibodies (IL-6 mAb) for the prompt detection of traumatic brain injury (TBI) by quantifying cytokine interleukin-6 (IL-6) in cerebrospinal fluid (CSF) [106]. Their sensor was tested using EIS and showcased excellent selectivity and LOD of 1.63 pg/mL.

#### 3.1.2. Carbon-Based IDEA

Highlighting the advantages of carbon as an electrode material, C-MEMS/C-NEMS is one of the emerging fabrication techniques in fabricating carbon-based IDEAs, due to the simplicity of the fabrication steps and the ability to produce a high aspect ratio IDEA. The carbon microelectromechanical systems (C-MEMS) and carbon nanoelectromechanical systems (C-NEMS) fabrication techniques allow the production of high aspect ratio of carbon structures via a conventional photolithography technique to pattern the desired shapes of photoresist polymers followed by pyrolysis [107]. As a result, electrodes with carbonized patterns can be easily fabricated according to desired structures and geometries. An epoxy-based photoresist, SU-8, is a good substance to fabricate high aspect ratio structures. SU-8 is a highly sensitive negative tone photoresist that exists in various viscosities, influencing the resultant thickness of patterned surfaces, ranging from hundreds of micrometers to submicron levels [108]. Due to the flexibility of the deposition thickness, SU-8 is one of the popular substances used to fabricate high aspect ratio structures using lithography methods. The lithography methods can be categorized into two types, namely masked lithography and maskless lithography. As reflected by the name, masked lithography method is used to transfer a pattern onto the substrate using a mask. Ultraviolet (UV) photolithography is one of the masked lithography methods [109,110]. Photolithography is a patterning method that uses UV light exposure to light-sensitive polymers (photoresist) to create the desired patterns. The illumination of UV light via an opaque feature of a photomask positioned on a transparent substrate creates an exposure on a photoresist, which is coated on a substrate [55,111,112]. Wang et al. pioneered the high aspect ratio C-MEMS devices with the ratio of 10:1 [113]. In later years, following the first report, many research works adapted a similar strategy to fabricate their C-MEMS and C-NEMS devices [114,115,116]. 

**Figure 3:**
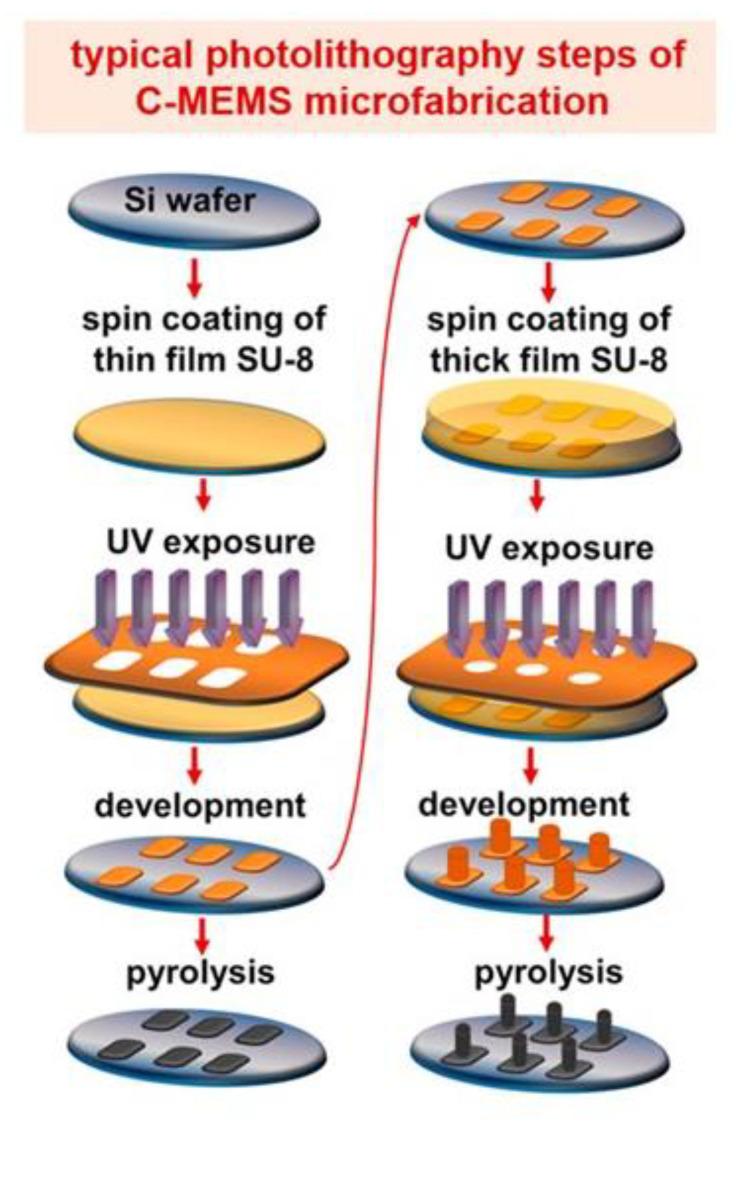
Schematic illustration of a typical photolithography process to fabricate 2D and 3D C-MEMS electrodes. Reprinted with permission from Ref. [118]. Copyright © 2022 Elsevier B.V. All rights reserved.

Figure 3 showcases the difference between the 2D and 3D designs of the C-MEMS electrode. The fabrication of 2D and 3D C-MEMS requires the photopatterning of the base layer (e.g., the IDEA) with a thickness ranging from 5–25 μm for 2D, while 3D requires the patterning of the second layer on top of the first layer. However, the second layer of the 3D pattern can be manipulated using the mask design. Then, the pattern is pyrolyzed under an inert atmosphere at temperatures above 600 °C to produce carbon structures. Pramanick et al. reported that the optimized temperature for electrochemical sensing applications is 900 °C [83]. In addition to the simplicity and fewer fabrication steps, the advantages of the fabrication techniques of C-MEMS and C-NEMS include low cost, good control of resistivity, and mechanical properties of carbon by varying the temperature of pyrolysis, controlling the porosity by varying the temperature ramp rates, and having high reproducibility [117]. 

As such, Liu et al. [119] compared the expected simulation response with the IDEA fabricated via C-MEMS technique. They compared five different IDEA gaps (g) between 2.7 μm and 16.7 μm, and widths (w) between 1.3 μm and 2.3 μm. They reported that the smallest electrode gap spacing of 2.7 μm and an electrode width of 1.3 μm recorded the highest steady-state currents in the generator/collector mode 4.7 μA (experimental) and 3.8 μA (simulation) at a scan rate of 10 mV/s, as well as a collection efficiency up to 98%, revealing that approximately all products from the generator have reached the collector. For larger IDEA gaps, they obtained quantitative agreement between simulation and experimental data, but for the smallest IDEA electrodes, they recorded larger currents than the predicted simulation. The authors concluded that this could be due to convection caused by electrokinetic flow. 

The combination of metal and carbon in IDEA have proved enhancement of the collection efficiency of IDEA-based electrochemical sensors. A follow-up study by Liu et al. [120] compared the CV performance of the carbon–platinum IDEA (C-Pt IDEA) with a carbon–carbon IDEA (C-C IDEA) [119], using homogeneous catalytic production of hydrogen (H_2_) as the test redox system [120]. The width of the carbon generator electrode was 2 μm (slightly wider because of overplating of the Pt), and the gap between two adjacent fingers was 3 μm. They found that in the same H_2_ production reaction, the C-Pt IDEA current was higher (collection efficiency 68%) than the C-C IDEA (collection efficiency 37%) in the presence of acid. However, they also observed that the combination of the C-Pt IDEA did not achieve higher collection efficiency in the presence of acid. Thus, their findings proved that the addition of Pt on C increased collection efficiency for the homogeneous catalytic production of hydrogen (H_2_).

### 3.2. Method to Increase Surface Area in IDEA-Based Electrochemical Sensor

In an effort to maintain the IDEA electrode measurement within nano to micro sizes, the sensitivity and specificity of 3D IDEA-based electrochemical sensors can be facilely controlled via manipulation of the IDEA geometries and structure, integration of microchannels, integration of nanoparticles, and fabrication of IDEA from various substances as electrode materials. Escalations in the height of 3D IDEA increase surface area and diffusion area in electrolytic solutions. As a result, redox amplification in electrodes increases and improves the sensor sensitivity. Furthermore, the sensitivity of the electrochemical sensor from IDEA is label-free and highly functional for practical biosensing applications. The selectivity can be determined by the labelling method between the target analyte and receptor in some cases.

#### 3.2.1. Microchannel Insertion

The electrochemical responses of the electrode with the integration of the microchannel are affected by several factors, such as solution flow rate, dimensions of the electrode and the channel, the potential sweep rate, etc. [121]. The miniaturization of the IDEA’s electrode dimension with channel encapsulation does improve the redox reaction but changes the radial diffusion on the IDEA. Additionally, Heo et al. [49] studied the simulation of 1:1 aspect ratio of two types of 3D IDEA nanoelectrodes that have similar width (w = 650 nm) and electrode gaps (g = 2.35 µm) but different heights (3D IDEA nanoelectrode (h = 650 nm)). They further investigated the electrode ratio effect on redox cycling and the influence of different microchannel heights (h = 1 to 10 µm) on a 3D IDEA thin-band nanoelectrode (h = 100 nm). Their team reported that the lowest height of the microchannel interrupted the radial diffusion to the electrodes and caused the decrease in diffusion to the top surface, thus, reducing the total current. Moreover, their simulated sample confinement also enhanced the collection efficiency up to 98%, which aligned with Ueno et al. [121,122] who reported that collection efficiency in the presence of a microchannel was higher than without a microchannel. 

The integration of the microchannel affected the electrochemical sensor performance and increased the IDEAs’ whole surface area too. As such, Heo et al. [49] fabricated carbon 3D IDEA nanoelectrodes from SU-8 negative photoresists using the C-MEMS method integrated into the polydimethylsiloxane (PDMS) microchannel for dopamine application. Interestingly, they found that chronoamperometry (CA) recorded the highest signal amplification at 1116 with a 10 µm high microchannel, when performed for the same duration as both single-mode and dual-mode currents. It was concluded that channel height caused higher dual-mode currents (3.38 mA), compared to single-mode current (3.03 nA), due to the sample confinement affected by the reduction in single-mode current. They also measured various concentrations (10 µM to 10 mM) of dopamine in 0.1 mol L^–1^ phosphate-buffered solution (PBS) to test the carbon biosensor applicability. The adsorption of dopamine caused large iR drops and lost linearity in the electrochemical current response when the biosensor was tested in high-dopamine solutions. Nevertheless, in low concentrations, the redox currents became well saturated, which means the carbon 3D IDEA nanoelectrodes surface was less vulnerable, compared to metal electrodes when the chemical reaction of dopamine occurred. Thus, the integration of the microchannel affected the electrochemical sensor performance and increases the IDEA’s whole surface area.

**Figure 4:**
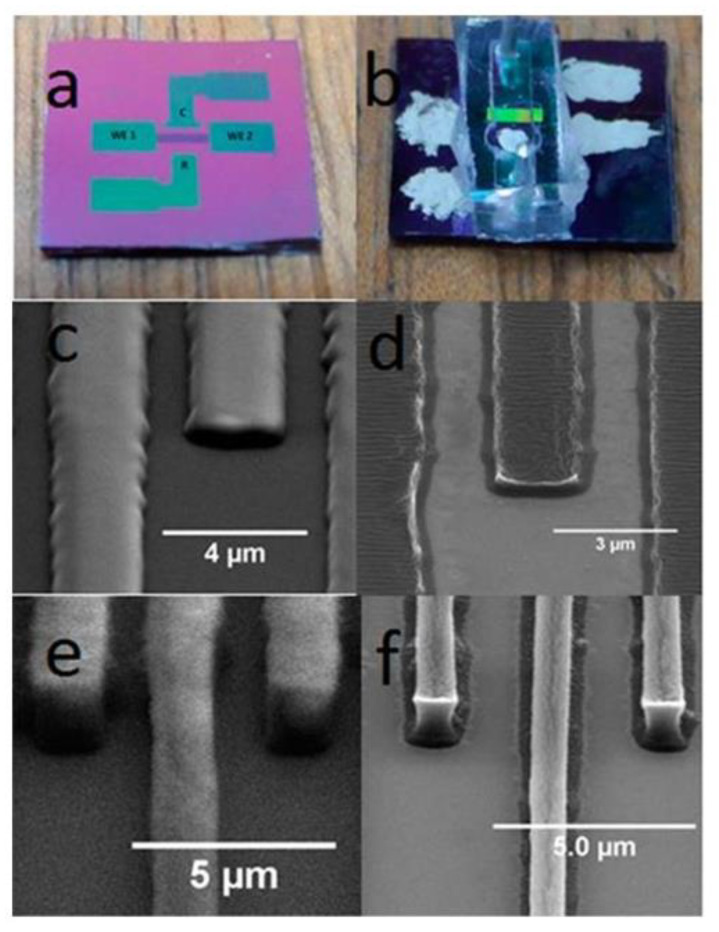
(**a**) 3D carbon IDEA sensor on a Si wafer. WE1 and WE2 are contact pads for the generator and the collector, respectively. C and R are counter and reference electrodes, respectively. (**b**) 3D carbon IDEA integrated with PDMS channels. The reference electrode is coated with Ag/AgCl ink, and contact pads are coated with silver paste for better electrical connection. (**c**,**e**) Scanning electron microscopy (SEM) images (tilted view 60°) under 10,000 × magnification of SU-8 IDEA patterning before pyrolysis; height = 0.6 and 2.1 μm, respectively. (**d**,**f**) Carbon IDEA after pyrolysis; height = 0.22 and 0.59 μm, respectively. Reprinted with permission from [54]. Copyright © 2022, American Chemical Society.

The study of flow and no flow conditions in microchannel does perturb the sensor sensitivity. Kamath et al. [54] presented the 3D carbon microelectrodes IDEA from low-viscosity negative photoresists (SU-8 2000.5, SU-8 2002, and SU-8 2005) material, fabricated using C-MEMS. They analyzed the sensor performance under flow and no-flow conditions using CV and CA. The 3D IDEA was enclosed in a PDMS channel (100 μm high and 1.5 mm wide), as shown in Figure 4, to ensure little evaporation of the solution during the experiment. Their study showed that an increase in 3D carbon IDEA height (height of 1.1 μm) and a width/gap ratio of 1.58 (w bottom = 2.7 μm, w top = 1.95 μm, gap bottom = 1.1 μm, and gap top = 1.85 μm), which increases the redox amplification factor to 37 with a collection efficiency of 98.6%. Their results showed that amplification dropped from 37 to 4 for the same IDEA electrode at a flow rate of 500 nL/s due to redox cycling hindered by the convection. Under no-flow conditions, an increase in the IDEA’s height caused higher redox amplification, whereas, under flow conditions, lower signal enhancement was being detected because the flow was detrimental to the elliptical diffusion between the horizontal edges of the 3D carbon IDEA. Their team focused on higher and wider IDEA’s height and width in µm unit, while their gap spacing used was smaller than Heo et al. [49], which proved the advantages of the smaller gap spacing in carbon IDEA’s design. However, further details on the flow and no-flow conditions in PDMS microchannel with the different heights of a microchannel affecting the redox amplification was not studied. The lower height of the microchannel (less than 100 µm) should be considered and incorporated for further studies of flow and no-flow conditions and their consequences on IDEAs’ sensor performance. 

#### 3.2.2. IDEA Geometry and Structures

The IDEA-based sensors have been extensively explored [123,124,125]. Several works have demonstrated that higher IDEA thickness or height of the electrodes results in higher redox amplification factors [49,54,126,127]. In addition, increasing the surface area of IDEA-based sensors can be achieved in various ways, such as by fabricating pillars on top of the 2D IDEAs [128,129], by controlling the spin-coated thickness and shape of the photoresist carbon precursor material [54,130,131,132,133,134], and by growing vertically aligned carbon nanotubes (VACNT) on top of planar IDEA [135,136]. Interestingly, without increasing the extent of the IDEA’s thickness, the integrated microchannel [121,137,138] and combination with a mesh [139] on IDEAs does improve the amplification factor due to the large surface area of the redox cycling event. In addition, the design of 3D IDEA sensors will also improve the sensitivity [140] and redox amplification/amplification factor, compared to planar IDEA sensors. 

Current research has shown that simulation and comparison of the 2D IDEA and 3D IDEA are indeed important to provide empirical results prior to the actual fabrication of 3D IDEA biosensors for targeted biosensing applications. The comparison between the simulation and actual experiment of 2D IDEA and 3D IDEA was published by Han et al. [141]. They studied four different systems of the geometric configuration effect of IDEA electrodes, which are Open-2D IDEA, Closed-2D IDEA, two straight electrodes in parallel, and 3D IDEA using the simulation process from COMSOL Multiphysics software. From these four patterns of geometric configuration, 3D IDEA simulation has the best result for electrochemical immunosensing. Subsequently, they fabricated the 3D IDEA using photoresist AZ4620 with modification to the indium tin oxide (ITO) electrode, alkaline phosphatase (ALP) as the enzyme label, electroactive ferrocene (Fc) as the electron mediator, and p-aminophenyl phosphate (pAPP) as the enzyme substrate. They demonstrated that 3D IDEAs consisted of a thin layer of solution narrowed between the two IDEA electrodes with a 5 μm width and 10 μm gap between the bottom and the ceiling height (several tens of µm), corresponding to the height of the microchannel. They also tested their biosensor using amperometric and chronocoulometric methods besides impedimetric detection. Their proposed fabrication technique also required no addition of biological additives. They tested the 3D IDEA immunosensor for mouse IgG and cardiac troponin I (cTnI). Based on their results, the 3D IDEA achieved an LOD of ∼10 fg/mL and ∼100 fg/mL for the Closed-2D IDA for detection limit in mouse IgG, whereas 3D IDEA obtained a limit of detection of 100 fg/mL for cTnI. Their findings proved that 3D IDEA did not only increase the surface area but also decreased the LOD values. 

**Figure 5:**
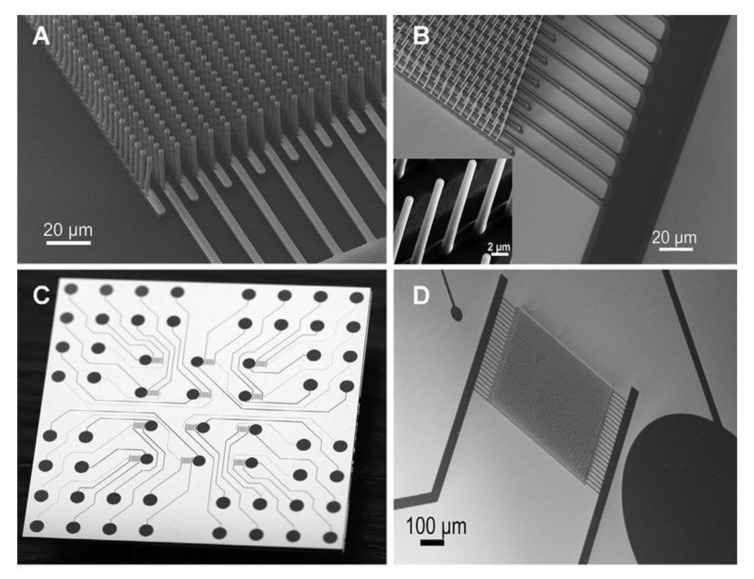
SEM images of 3D IDEAs with carbon pillars (diameter of 1.4 µm, height of 11 µm) interconnected by interdigitated structures: (**A**) before pyrolysis; and (**B**) after pyrolysis. (**C**) Photograph of a silicon chip with the pyrolyzed carbon electrode array structures (12 three-electrode systems at the center with surrounding contact pads). (**D**) SEM image of a three-electrode system. Reprinted from [129], Copyright © 2022 Elsevier Ltd. All rights reserved.

Moreover, shrinkage is one of the factors that changed the height during the pyrolysis step. In addition to the height or thickness of spin-coated materials building up into one solid rectangle shape for the whole width of the IDEAs’ finger array during the photolithography technique, the modification of IDEA geometry into a pillar-shaped array and onto the IDEA’s fingers transforms the whole shape of the basic 3D IDEA pattern. This has been reported by Amato et al. [129]. Their team demonstrated a high aspect ratio of 3D carbon pillars synthesized from SU-8 2075 on top of SU-8 2005-based planar 2D carbon IDEAs. Figure 5 illustrated the C-MEMS fabrication process using the negative photoresist materials followed by analysis via CV and EIS for further investigation of their sensor performance. They successfully fabricated 3D carbon pillars with 1.4 µm diameter, a centre-to-centre spacing of 5 µm, and an aspect ratio of about 8 and 11 µm in height. Their team also described the shrinkage of planar 2D IDEAs that interconnects the pillars yield carbon digits (a decrease of 91.7 ± 0.5% vertically) and width (a decrease of 27 ± 3% laterally) from what was originally 5 µm width and thickness. The 3D carbon pillars shrunk to 1.4 µm in diameter and 11 µm in height. The pyrolysis process resulted in a loss of material caused by the evaporation of CO_2_, hydrocarbons, and other gases during photoresist decomposition and aromatization, leading to the shrinkage of the structure [142]. Their team achieved a high aspect ratio 3D IDEA that produced a quasi-reversible CV with a peak potential separation (ΔEp) value of 168 ± 12 mV. Their presented 3D IDEA’s pillar array successfully increased the surface area up to 70%, compared to the same electrode without pillars.

Another type of 3D carbon pillar was mentioned by Bose et al. [143], whereby their team fabricated a 3D carbon IDEA in which their 3D pillar expressed a shape that was similar to that of Amato et al. [129]. In contrast, the diameter of their pillar was 20 µm on top of the 2D IDEA. They spin-coated the SU-8 2000 on quartz as the substrate, followed by infrared lithography and pyrolysis. Their conductive carbon-based capacitive IDEA sensor exhibited a sensitivity value of 2.741 µA mM^–1^ cm^2^ for glucose testing. The difference was that they used quartz as a substrate instead of silicon. Therefore, these finding highlighted the importance of a substrate in the fabrication of functional electrodes [144].

**Figure 6:**
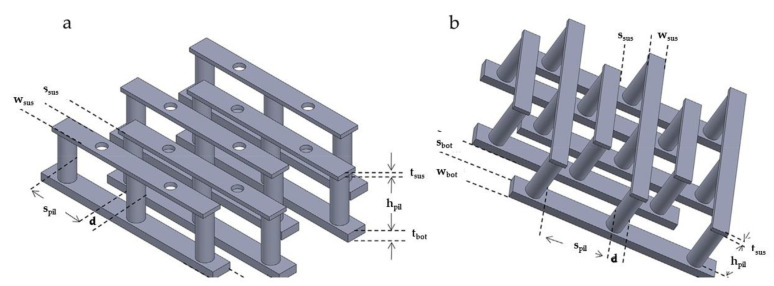
Suspended layer with: (**a**) stripes connecting pillars of the same finger (3D//); and (**b**) stripes connecting different fingers of the same electrode (3D_#_). Reproduced from [145]. Copyright © 2022, Elsevier under the terms of the Creative Common CC-BY license.

The aforementioned carbon 3D IDEA design showed a clear boundary between collector and generator fingers with enhanced surface area. The challenging elements of increasing the surface area of carbon 2D IDEA to 3D IDEA have been explored by adding the suspended beam on top of the existing pillars forming a unique 3D IDEA architecture (see Figure 6). Interestingly, Mantis et al. [145] fabricated a complex IDEA from the carbon-based 2D IDEA to the novel suspended carbon 3D IDEA using three types of photoresists via C-MEMS technique and tested their electrodes using CV and EIS. Their team fabricated 3D interdigitated electrodes with pillars on different fingers connected through suspended interdigitated microstructures, as shown in Figure 6a,b. The bottom part composed of 2D IDEA was synthesized from SU-8 2035. The micropillars were fabricated from SU-8 2075, whereas the suspended layers consisted of mr-DWL-40 photoresist. Their team successfully increased the 3D IDEA surface area by adding the suspended IDEA supported with micropillars onto the 2D IDEA pattern. Both the fingers of interdigitated electrodes were used as an electrode in which one of the interdigitated carbon electrodes suspended carbon IDEA dimensions; 2D_p_-25, 3D_#_-25 bottom IDEA and pillars: w_bot_ = 25 µm, s_bot_ = 25 µm, t_bot_ = 17 µm, d = 20 µm, s_pil_ = 60 µm, h_pil_ = 100 µm, and 3D_#_-25 suspended IDEA: w_sus_ = 10 µm, s_sus_ = 20 µm, t_sus_ = 17 µm. The highest anodic steady-state current, I_p_ = 0.527 ± 0.003 mA, was achieved by 3D_#_-25, while for 2D IDEA, the 2D_p_-25 recorded highest I_p_ = 0.23 ± 0.02 mA. Their results proved that 3D_#_-25 increased the surface area of the electrode as it recorded a three times larger CV peak current, compared to the 2D electrode, 2D_p_-25. Their findings emphasized that the complex was used as WE and the other one as CE for both CV and EIS. The 3D-suspended carbon 3D IDEA was a method used to increase the IDEA’s surface area, improving the sensor performance, compared to the original carbon 2D IDEA.

**Figure 7:**
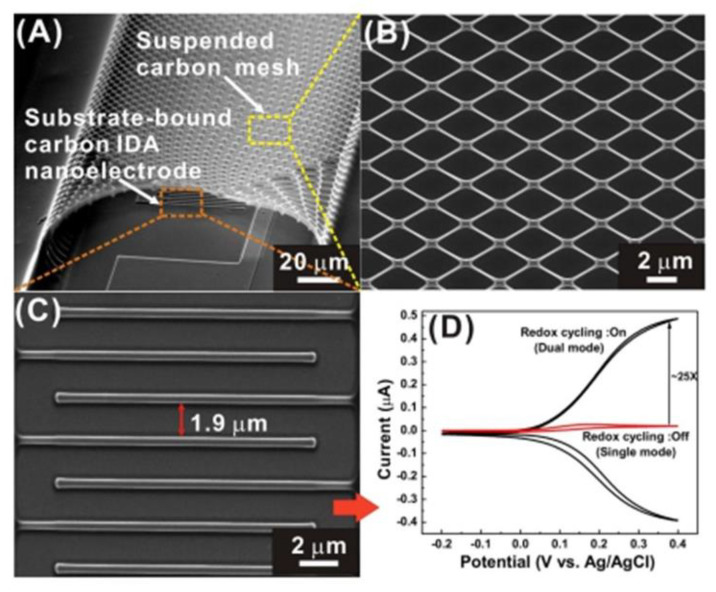
(**A**) SEM image of a 3D carbon system (tilted-view); (**B**) enlarged top-view images of a suspended carbon mesh; (**C**) substrate-bound carbon IDEA nanoelectrodes; and (**D**) cyclic voltammograms of 1 mM PAP in 0.1 M PBS at the IDEA nanoelectrodes. Both the combs were scanned from −0.2 to 0.4 V vs. Ag/AgCl in the single-mode (red line). In the dual mode, the potential of the generator comb was scanned the same as the single-mode while the collector comb was held at −0.3 V (black line). Amplification factor (AF) = sum of dual-mode current from IDEA/sum of single-mode current from IDEA. Reprinted from [139]. Copyright © 2022 Elsevier B.V. All rights reserved.

Another interesting piece of research to increase IDEA sensor performance while retaining the IDEA original structural layer was conducted by Sharma et al. [139] in which they added the mesh-like shape on top of a carbon 3D IDEA. Continuing the IDEA fabrication process using C-MEMS, they produced a 2 μm thick SU-8 2002 positive photoresist (AZ P4330) coated on top of a pre-patterned carbon IDEA, followed by the spin coating of 18 μm thick SU-8 2025. The unique IDEA fabrication method of using a small UV dose through a mesh-shaped photomask successfully facilitated the shallow polymerization of the top layer of spin-coated photoresist. A 5 μm thick suspended polymer mesh supported by two 18 μm thick posts was achieved after the development of a double exposed negative photoresist followed by pyrolysis. The substrate-bound IDEA gap of ~1.9 µm and a few micrometers apart from the suspended carbon mesh (width ~300 nm) are shown in Figure 7. The CV dual-mode resulted in high signal amplification of ~25 from redox cycling of PAP/PQI, compared to single-mode CV. Their 3D carbon immunosensor recorded a linear detection range of 0.001 to 100 ng/mL for cardiac myoglobin (cMyo). The immunosensor successfully recorded a low detection limit of 0.43 pg/mL cMyo in phosphate-buffered saline and human serum [139]. Their distinctive method for improving the IDEA-based sensor proved that the C-MEMS technique is not only limited to the simple fabrication of 3D IDEA design but is also capable of producing a distinct mesh shape. 

#### 3.2.3. Three-Dimensional (3D) IDEA in a Two-Electrode Configuration

**Figure 8:**
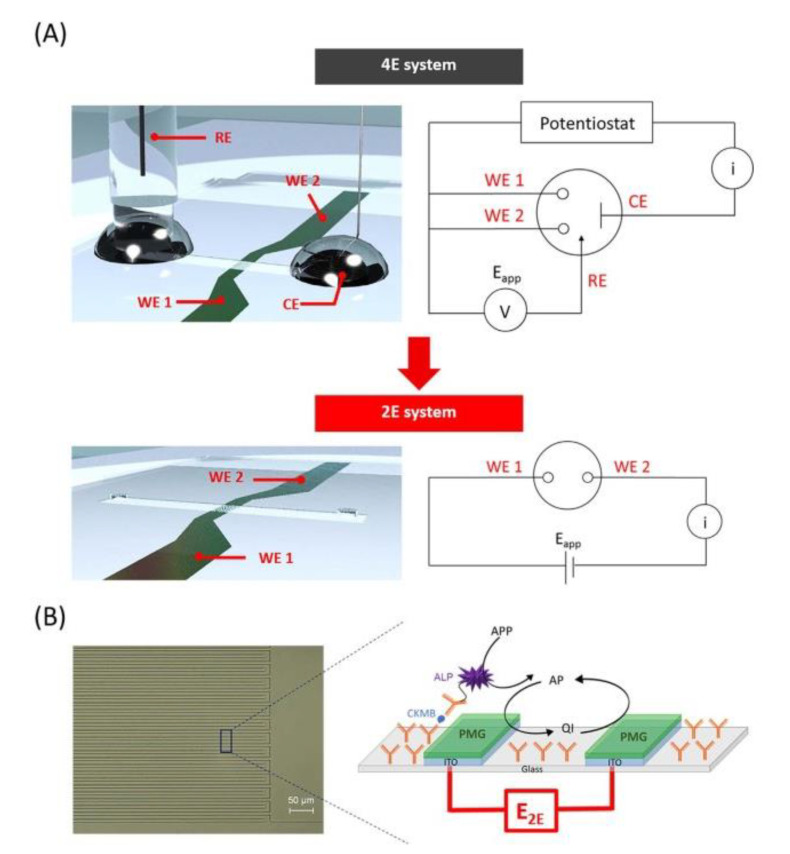
(**A**) Scheme of four-electrode (4E) and two-electrode (2E) systems in IDEA microchip; (**B**) optical microscopic image of IDEA and schematic representation of electrochemical immunoassay in IDEA microchip. Illustrations of cross-sectional view of IDEA surface showing immobilization of antibodies onto a glass substrate, where ITO is etched, followed by immobilization of PMG with dopamine (DA) onto ITO. Reprinted from Ref. [146]. Copyright © 2022 Elsevier B.V.

Generally, IDEA’s four-electrode configuration requires a reference electrode and a counter electrode. Interestingly, Lee et al. [146] eliminated the reference electrode and counter electrode in their IDEA-based electrochemical sensor, leaving the IDEA integrated into the microchannel to increase the surface area. The research team fabricated the 3D IDEA using a similar method as that published by Han et al. [141] albeit with modifications. They modified the four-electrode (4E) system (consisting of two working electrodes, the counter and reference electrode) to a two-electrode (2E) system, alternatively aligned with a 5 μm wide, 10 μm gap between adjacent fingers, and a height that depended on the height of the channel (30 μm) without the counter and reference electrode, as shown in Figure 8 [146]. They successfully established their IDEA working principle by introducing a redox mediator film, poly(methylene green) (PMG), immobilized with poly(dopamine) (PDA) onto the indium tin oxide (ITO) 3D IDEA chip configuration by electropolymerization. Uniquely, one of the working electrodes was used to observe the electrochemical signal, whereas the other working electrode worked simultaneously as counter and reference electrodes in which oxidation and reduction occurred. This is an interesting piece of work, compared to the previous carbon 2D IDEA and 3D IDEA. In this research, they further tested the 2E system of ITO 3D IDEA with human creatine kinase-MB (CK-MB) and a detection limit of 0.32 pg/mL was achieved, confirming the fabrication of reliable and highly sensitive electrodes. The advantage of their two-electrode IDEAs system was that any electrodes can be fabricated from the same starting materials, resulting in a less complicated fabrication process. In future, their distinctive IDEA testing without reference and counter electrodes can be further explored and may be applied for carbon IDEA with some modifications to the carbon surface. In addition, Table 2 summarized the comparison between the carbon-based 2D IDEA (2D C-IDEA) and carbon-based 3D IDEA (3D C-IDEA) sensor performance using CV based on amperometric sensing technique.

## 4. Nanocomposites IDEA

### 4.1. Vertically Aligned Carbon Nanotube (VACNT)

**Figure 9:**
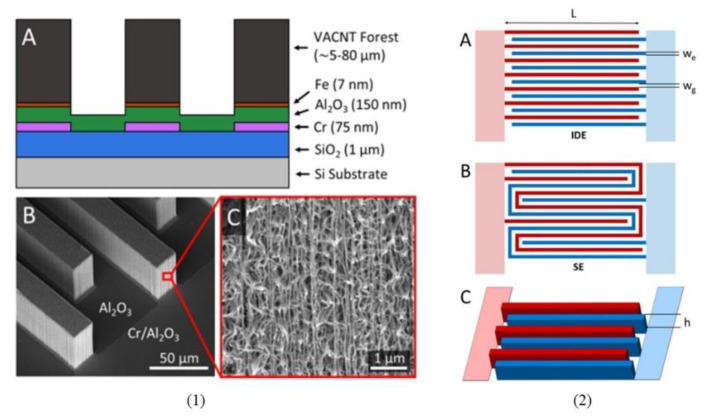
(1) (**A**) Schematic of layers used to fabricate the VACNT sensor architecture: Si, SiO_2_, Cr, Al_2_O_3_, Fe, and VACNTs; (**B**) scanning electron microscopy (SEM) images of 3D VACNT electrodes; and (**C**) magnified VACNTs, showing the porous nature of an electrode. (2) Schematic of the: (**A**) IDE; and (**B**) SE electrode arrangements with electrode length (L), electrode width (we), and gap width (wg) represented. Red and blue distinguish the different electrodes in the sensor, with the dark colors representing regions of VACNTs and light colors representing Cr leads under Al_2_O_3_. (**C**) Schematic emphasizing the 3D nature of VACNT IDEA electrodes with electrode height (h) represented. Reprinted with permission from [136]. Copyright © 2022, American Chemical Society.

In addition to the modification of carbon 3D IDEA’s height structures, another method for increasing the IDEA’s surface area and sensor sensitivity is through the integration of porous vertically aligned carbon nanotubes (VACNT) onto the IDEA design. Brownlee et al. [136] investigated the IDEA design surface area by fabricating a porous 3D VACNT IDEA and comparing it with a serpentine electrode using CV and EIS, as shown in Figure 9. They fabricated four electrodes of 3D VACNT IDEA and two serpentine (SE) electrodes where the width and gap for 3D VACNT IDEA and SE were similar; the width of the electrode (w = 20 μm and 25 μm), the gap between adjacent fingers (gap width, g = 15 μm and 25 μm), and height for 3D VACNT IDEA (h = 5 μm, 25 μm, and 80 μm) and SE (h = 80 μm). Their fabrication method did not employ the C-MEMS method; instead photolithography was used to pattern the positive photoresist AZ3330 with Fe as the substrate. The growth of VACNT followed published protocols [147,148]. Based on CV and EIS analyses, they found that the 3D VACNT IDEA (h = 80 μm and g = 15 μm) resulted in 1.6 times higher sensitivity than SEs and had 4.3 times higher sensitivity, compared to the 5 μm height 3D VACNT IDEA. The biosensors were then tested with streptavidin and biotin. The 80 μm height of the 3D VACNT IDEA with a gap of 15 μm demonstrated an LOD of 1 ng/mL F-biotin, equivalent to other reported work [149,150]. It has been found that the promising electrodes expressed the highest sensitivity, most linear-sensing regions, and an electroactive surface area of 15 times higher than the 2D geometric area. These findings showed that the integration of VACNT on metal IDEA structure can potentially enhance the biosensor’s sensitivity.

In a similar piece of work, Ding et al. [151] used silicon/silicon oxide wafer, AZ3330 resist, and iron catalytic layer as the IDEA base to fabricate 3D VACNT IDEA (labeled as VANTAs). However, the modification performed by Ding and colleagues focused on the height-to-width ratio of 3:1, whereas Brownlee et al. [136] compared 3D VACNT IDEA (different w, g, and h dimensions) with the serpentine electrodes. They reported a 2D IDEA pattern for their immunosensor CIP2A using non-carbon electrode material followed by testing using CV. In situ amorphous carbon infiltration into the CNT forest was performed to produce robust and porous arrays of IDEA fingers. These immunosensor exhibited the detection of label-free CIP2A across a wide linear-sensing range (1–100 pg/mL) with an LOD of 0.24 pg/mL within saliva supernatant without the need for sample pre-labelling or pre-concentration methods. Moreover, the faradaic EIS detection method used for these immunosensors did not require a three-electrode electrochemical setup or reference electrode to make the VACNT IDEA fit for mass fabrication, miniaturization, and integration into microfluidic channels [151,152]. They also reported that the approximate 1% of active sites at a scan rate of 50 mV/s in CV was greater than the approximate 0.4% of active sites from previous reports [153]. Hence, the porous architecture of VACNT IDEAs is advantageous in elevating the electroactive surface area beyond a conventional solid or planar IDEA sensor [154], and it also offers better active sites or carbon–carbon defects, compared to conventional CNT electrodes [151,155,156]. 

### 4.2. Nanoparticles

Nanoparticles are particles ranging between 1 and 100 nm and are typically categorized into organic, inorganic, and carbon-based nanoparticles [157,158]. Different compositions and sizes of nanoparticles exhibit different functions, and hence can be adapted for specific electrochemical sensings, such as immunosensors, enzyme sensors, etc. [151,155,156]. Organic nanoparticles, such as micelles, liposomes, and dendrimers, are common organic nanoparticles with non-toxic and biodegradable properties. Nanocapsules (e.g., micelles and liposomes with hollow cores) are sensitive to thermal and electromagnetic radiation, such as light and heat [159]. Despite that, organic nanoparticles are popular in biomedical fields, especially drug delivery systems, because of their efficiency and ability to be injected into specific body parts [160]. 

Metal-based nanoparticles are inorganic nanoparticles produced from metal-based materials via constructive or destructive methods and practically almost all metals can be synthesized into specific nanoscale ranges [161]. To date, metal-based nanoparticles from gold (Au), iron (Fe), lead (Pb), aluminum (Al), cadmium (Cd), cobalt (Co), silver (Ag), and zinc (Zn) have been extensively studied for various biomedical applications [162,163,164,165,166]. Notable characteristics of metal-based nanoparticles include crystalline and amorphous structures, high surface area to volume ratio, pore size, surface charge density, sensitivity, and reactivity to environmental factors, such as sunlight, air, moisture, heat, etc. [167]. Metal nanoparticles are generally used as “electronic wires” to enhance electron transfer between an electrode’s surface and redox centers in proteins because they have good conductivity. Moreover, these nanoparticles also present a good catalytic characteristic as promising catalysts for improving and increasing electrochemical reactions. For instance, Au nanoparticles or AuNP were integrated into IDEA by Sharma et al. [168]. However, Sharma and colleagues reported that one of the downsides of incorporating AuNPs into IDEA was a corrosion problem upon contact between the metal-based nanoparticles and electrolytes, confirmed via CV. In this case, considerable attention has been directed toward integrating carbon with metal electrodes for IDEA to curb the aforementioned problem. 

Metal oxide-based nanoparticles [169] are developed to tackle the drawbacks of metal-based nanoparticles. For instance, in the presence of oxygen at room temperature, iron oxide (Fe_2_O_3_) is oxidized from iron (Fe) nanoparticles, thus, increasing its efficiency and reactivity, compared to iron nanoparticles [7]. Examples of common metal oxide-based nanoparticles are iron oxide (Fe_2_O_3_), zinc oxide (ZnO), magnetite (Fe_3_O_4_), silicon dioxide (SiO_2_), aluminum oxide (Al_2_O_3_), cerium oxide (CeO_2_), titanium oxide (TiO_2_), and many more [170,171,172]. As such, magnetic iron oxide nanoparticles (IONPs) have been used for tumour-targeted gene delivery [173], owing to their propitious properties, such as ease of chemical functionalization, high biocompatibility, low toxicity, direct synthesis methods, and superior magnetic responsiveness [174,175,176,177]. 

Despite the mentioned advantages of carbon-based IDEA, its electrical conductivity is slightly lower than most metal-based IDEAs [178], resulting in a lower electrochemical performance. Therefore, complementary materials, such as gold nanoparticles (AuNPs), may help to enhance biosensor performance by facilitating the electron transfer and conductivity of the electrode to increase analytical selectivity and sensitivity. Gold nanoparticle-based IDEAs provide great advantages, such as chemical stability, quantum size effects, and ease of synthesis advantageous for electronics, catalytic, and optical characteristics. Moreover, the high surface-to-volume ratio of AuNPs increases several magnitudes in electroactive surfaces, leading to high sensitivity and higher enzyme loading within the integrated devices [179]. 

The addition of AuNPs to IDEA has been studied by Sharma et al. [168] in which they dispersed the AuNPs on top of a 3D carbon IDEA. Furthermore, the fabricated glassy 3D carbon IDEA (3D C-IDEA) was composed of SU-8 2002 on SiO_2_/Si wafer achieved via C-MEMS process with an aspect ratio of 1:1 to obtain 620 nm width, 650 nm height, and a gap between the adjacent comb of 1.9 µm. The fabricated AuNP/3D C-IDEA was prepared for cholesterol detection; hence, cholesterol oxidase (ChOx) was selectively immobilized on the AuNP/3D C-IDEA via electrochemical reduction of the diazonium cation. They analyzed the sensitivity of the AuNP/3D C-IDEA-based cholesterol biosensor via amplification of redox mediators between the combs of AuNP/3D C-IDEAs and immobilized enzymes area. The results were categorized as functionalized and non-functionalized with a wide sensing range (0.005–10 mM). The AuNP/3D C-IDEAs biosensor recorded lowest the LOD value (~1.28 µM) with high sensitivity (~993.91 µA mM^–1^ cm^–2^), as compared to bare carbon IDEAs with an LOD of 4.15 µM and a sensitivity of 790.75 µA mM^–1^ cm^–2^. Thus, the alteration of carbon IDEA with AuNP demonstrated enhanced sensor sensitivity and lower LOD values too.

**Figure 10:**
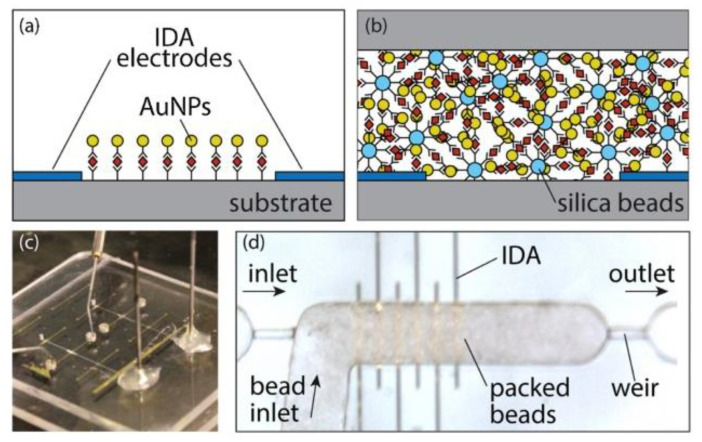
Idealized views of: (**a**) planar; (**b**) a volumetric impedimetric immunosensor; (**c**) fabricated thermoplastic device; and (**d**) magnified view of the detection zone, including thin-film gold IDEA and packed bed of functionalized silica beads in a 150 µm deep channel. Reprinted from [180], Copyright 2016, with permission from Elsevier.

The exciting combination between metal-based IDEA and nanomaterials towards innovative biosensing platforms stimulate an interesting piece of research led by Wiederoder et al. [180]. In this work, the research group prepared IDEA patterns with a combination of Cr/Au IDEA and porous sensor elements in the microfluidic channel. The preparation of the porous sensor in this work involved the functionalization of a packed bed of silica beads with antibody probes within a thermoplastic microchannel. The porous sensor element was completed with gold-based IDEA for further analysis by measuring the impedance changes in the concentration-dependent formation of silver aggregates, as illustrated in Figure 10. The 10 µm wide electrodes spaced 15, 40, and 100 µm apart within a 150 µm deep channel of IDEA’s sensors were tested with silica beads functionalized with anti-human rabbit IgG to determine the best electrode geometries. According to their test results, the spacing of 100 µm was shown to be the best IDEA for further experiments. They also conducted an immunoassay test by introducing the AuNP anti-rabbit conjugates followed by silver enhancement. Based on the impedance measurement results, all positive test samples were confirmed through the changes of a white porous silica bead bed to a red-brown color, which was also in agreement with previous studies [181]. Their fabricated device with low-resolution electrodes expressed a detection limit, ranging between 1 and 10 ng/mL with a 4-log dynamic range for sandwich immunoassay and total assay time of 75 min. The limit of detection of 10 ng/mL for the IgG model system was achieved. Compared to the planar biosensors that require serial functionalization of individual devices, the silica beads used in this experiment are advantageous in terms of off-chip functionalization for greater manufacturability. In addition, it offers the possibility of using the packing of multiple beads for the development of various target probes.

### 4.3. Summary of IDEA-Based Electrochemical Sensors

IDEA-based electrochemical sensors have been reviewed in many aspects due to their popularity. As such, Afsarimanesh et al. elaborate on planar IDEA sensors linked to many types of signal-conditioning circuits and their application in biomedical, environmental, and industrial sectors [21]. Forouzanfar et al. discussed the technical aspect of the devices fabricated using C-MEMS and C-NEMS from photolithography and non-photolithography techniques, specifically for biotech applications [118]. Brosel-Oliu et al. reviewed the IDEA using an impedance technique for a variety of bacteria detection explicitly in terms of bacterial growth monitoring and label-free specific bacteria [12]. Moreover, carbon-based microelectrodes for neurotransmitters have been published elsewhere [182]. Following extensive reviews by others, the practical application of IDEA-based electrochemical sensors, concentrating on unique IDEA structures as a method to increase the surface area and sensitivity of the sensor while maintaining the miniaturized size are presented in Table 3.

## 5. Challenges and Future Directions

The COVID-19 pandemic has shown that our world is very susceptible to the chaos caused by biological threats, notwithstanding the innumerable improvements in biosensor technologies every year. Current rapid biosensors still suffer from shortcomings concerning sensitivity and specificity for accurate detection and analysis. Despite numerous publications on IDEA-based biosensors with various widths, gaps, and heights fabricated from several fabrication methods, these exciting biosensing devices are still in need of improvements to circumvent several limitations. In this literature, we summarized several challenges and future directions for upcoming IDEA-based electrochemical sensors. 

### 5.1. Applications of IDEA-Based Sensors

Several ways to improve the IDEA-based electrochemical sensor performances have been discussed in the previous section, such as increasing the surface area of IDEA, the integration of nanocomposites, nanoparticles, and many more. In addition to the IDEA-based electrochemical sensors, Table 4 listed some applications of IDEA-based sensors between 2021 and 2022 to showcase the integration of IDEA with various materials to enhance the sensor performances for numerous applications.

### 5.2. Electrode Material

One of the struggles with fabricating the desired IDEA-based electrochemical sensors is related to the materials. Material selection to produce IDEA is vital because each material exhibits distinctive properties. The prerequisite characteristics of the best materials for IDEA should comply with the IDEA’s application paradigms. Up to now, research into the electrode materials of metal or carbon-MEMS IDEA-based electrochemical sensors has been extensively directed toward enhancing sensor sensitivity and selectivity. Metal/noble metal has been proven to exhibit excellent electrical conductivity, compared to pure carbon. However, some of the shortcomings of the noble metal include: (1) exhibiting unwanted electrochemical side reactions even at low potentials, and (2) corrosion upon contact with an electrolyte, for instance, Ti–Au or Cr–Au bilayers generate a galvanic couple and eventually corrosion [97]. In addition, some of the common noble metals for IDEA, e.g., Pt and Au need an adhesion layer, such as Ti or Cr, due to their weaker adhesion property onto Si or glass substrates. Nevertheless, metallic IDEAs have shown higher amplification factors, in contrast to other substrates, as presented in Table 3. Although Au possesses many advantages, such as high reliability and excellent electrical conductivity, it is not cost-effective and is easily diffused into substrates at low temperatures [94]. 

One of the main challenges in producing high-quality carbon IDEA is that poor conductivity of carbon may result in a large voltage drop. This hinders the miniaturizing of carbon MEMS electrodes to submicron ranges as resistance became overwhelmed in a small feature dimension [45,194,195,196,197]. Another challenge is the weak adhesion interface between the carbon layer and the substrate [45,195,197]. From this review, the integration of carbon and metal for IDEA’s electrode has been explored for a better sensor performance [120,168] to ultimately achieve a higher amplification factor and collection efficiency and lower limits of detection while maintaining the miniaturized size of IDEA-based sensors.

### 5.3. Optimization of the Fabrication Process

Among the fabrication methods of IDEAs, C-MEMS offers one of the simplest fabrications with fewer steps of photolithography followed by pyrolysis. Despite that, the price of devices fabricated using this technique is not cost-effective for the end users. Mass production of metal-based IDEAs can be challenging due to complicated fabrication processes. In this case, C-MEMS is better candidate due to the simplicity of the fabrication steps. In addition to the fabrication techniques, the standardization of process parameters plays an important role in the sensitivity and effectiveness of the carbon IDEA-based sensor prototypes for reproducibility. Mamishev et al. mentioned that environmental working conditions, such as acidity, economic value, temperature, pressure, and the drawback of parameter estimation algorithms, may limit certain electrode structures [198]. For example, methods for fabrication processes, such as UV photolithography [199] and pyrolysis [200,201], require the optimization of exposure time and temperature, which will influence the final electrode properties, etc. Chemical or physical vapor deposition is one of the high-cost methods for metal deposition. In addition to metal deposition, the lift-off step that followed is expected to leave metal residue on the substrate surfaces [97]. Thus, micrometers/sub-micrometer parameters that can affect the biosensor performance should be controlled for the large-scale production of biosensors [21,49].

### 5.4. Modification of Planar IDEAs to 3D IDEAs

This review reveals that the modification of 2D IDEA to 3D IDEA is not limited to increasing thickness to obtain the desired height of the IDEA’s fingers. Interestingly, increasing the surface area of the entire 3D IDEA has resulted in increased sensitivity of the sensor, which is a good indicator for building up an electrochemical sensor for various applications. In addition, the research on 2D and 3D IDEAs are expanding and focusing on the improvement of their performance and sensitivity while still maintaining micrometer or nanometer dimension. Current research is focusing on modifying and increasing the 3D IDEA surface area in order to obtain the higher sensitivity. Furthermore, the integration of microchannel as a method to increase the sensor surface area is quite exciting due to the different heights affecting the overall sensor reading. Studies related to the electrical double layer with or without the presence of the microchannels and flow or no-flow conditions within IDEA’s sensing space require detailed analysis not only limited to the source of the IDEA’s electrode materials but also the channel material [202,203].

Conversely, the build-up of 3D IDEA’s space can be explored further through the study of the interaction between its unique design, such as suspending 3D IDEA with a combination of different metal electrode materials because the materials orchestrate distinctive characteristics upon contact with electrolytes or application tests. Fascinatingly, the two-electrode system (2E) from IDEA’s design without the presence of the reference and counter electrodes, unlike the four-electrode configuration system, exhibit comparable results. Thus, further study on the IDEA’s width, gap, height, expanded surface area, integration of nanoparticles, suspended carbon mesh, metal IDEA base layer, and many more for the purpose of producing carbon 3D IDEA are recommended for future works. 

### 5.5. Reproducibility and Commercialization of IDEAs

Even though IDEAs are widely used in research for various advanced biosensors/sensors, limited number of promising sensors reached commercialization. One of the hindrances of transferring rapid biosensor technology from the lab to the commercial setting is batch-to-batch reproducibility. As the physiological samples are widely different, it is important to prevent the biosensor from reacting with other molecules from the samples using blocking agents. In addition, improvement of the sensor reproducibility for commercialization must focus on the automation of material handling. Automation allows full control of the equipment, particularly in the setting and production of the materials in a large scale production, e.g., the mixing process of high viscosity SU-8 2100 with cyclopentanone. Reagent dispensing using robotic pipettes onto the electrodes, etc., at the commercialization stage should be prioritized. The vast and fast production of small-sized sensors require accuracy at every fabrication stage. For example, the use of robotic pipettes may help to expedite the whole production process with minimal-to-zero dispensing errors. A controlled cleanroom facility for medical device manufacturing is the highest priority for upscaling manufacturing. Proper rules, regulations, frequent inspections by certified authorities for the overall manufacturing process should be implemented according to international laws. The regulations guidance for cleanroom space and the types of equipment are important for sensor/biosensor standardization and effectiveness in any application. It is also important to assess the global environmental impacts of mass production of biosensors, despite strict compliance with the standard protocols. Moreover, the integration of advanced telerobotic technologies for biosensor application testing will also have a huge impact on human healthcare as biosensor testing (e.g., dangerous viruses, such as COVID-19) can be performed remotely. Thus, the focus on the reproducibility and sustainability of IDEA biosensors is essential for successful commercialization.

## 6. Conclusions

The fabrication of IDEA-based electrochemical sensors is an evolving area of research because the biosensors/sensors configurations from three-electrode systems to four-electrode systems can be easily tailored via IDEA configuration. The unique design of IDEA makes it possible to be manipulated from planar IDEA to 3D IDEA via one of the simple fabrication methods, such as C-MEMS. The carbon-based MEMS method is straightforward, easy to fabricate, cost-effective, and reproducible. Furthermore, the carbon IDEAs based on carbon MEMS possess good criteria, such as high biocompatibility, good physicochemical characteristics, excellent chemical resistance, and stability for electrochemical measurements. This leads to high redox cycling efficiency due to the controllability of the carbon aspect ratios [50,83,204]. Carbon 2D IDEA is limited to low-signal amplification, unlike carbon 3D IDEA and metal IDEA/3D IDEA. The advantages of metal-based IDEAs include having very high amplification factors despite complex fabrication steps, compared to the C-MEMS method. A vast number of publications on IDEA-based electrochemical sensors reported a variation in 3D IDEA in terms of the width, height, and gap between adjacent fingers dimension, all of which increase the sensitivity of the sensors. Interestingly, in this review, several methods for increasing the 3D IDEA’s surface area are highlighted, such as microchannel insertion, unique IDEA’s structure (e.g., carbon pillar, the suspended carbon), VACNT, nanoparticles integration using MEMS and other methods. Moreover, the integration of mesh and microchannel on IDEA constrains the electrolyte in a limited space while retaining the good electrical double layer effect in the overall IDEA’s space. The combination of the carbon and metal/nanoparticle on the IDEA’s structure may also escalate the resultant surface area and sensitivity of IDEA-based electrochemical sensors. The increase in the IDEA’s surface area has shown better sensor sensitivity, compared to the basic 3D IDEA as it not only increases the height of the 3D IDEA but also expands the space for redox cycling. Moreover, miniaturized IDEAs are compatible with lab-on-chip devices for point-of-care testing systems. Despite the absence of reference and counter electrodes, IDEA’s modification as a two-electrode system presented comparable results to the four-electrode configuration system, proving that the IDEA’s unique design can be explored further for future IDEA-based electrochemical sensors. The authors of this review believe that the modification of carbon–IDEA designs with combination of carbon and metal IDEA-based electrochemical sensing techniques would be an ideal future research direction. These carbon–IDEA modification designs can deliver rapid and highly sensitive detection to chemical sensors for applications in food safety [205], homeland security [206], biomedical applications [207], environmental sensing [21], and industrial applications, among many others.

## Figures and Tables

**Table 2:** Comparison between carbon-based 2D IDEA (2D C-IDEA) and carbon-based 3D IDEA (3D C-IDEA) sensor performance using CV based on amperometric technique.

Electrode C-IDEA Sources	Dimension	IDEA Structure	Sensor Performance	Ref.
SU-8	w = 650 nm, h = 650 nm, g = 2.35 μm	3D C-IDEA	(1) AF = 10.8, CE = 96.8% in bulk solution (2) AF = 139 in h = 6 mm channel, (3) AF = 230 in h = 10 mm channel	[49]
SU-8 2000.5, SU- 8 2002, SU-8 2005	h = 1.1 μm and a w/g ratio of 1.58 (w bottom = 2.7, w top = 1.95, g bottom = 1.1 μm and g top = 1.85 μm)	3D C-IDEA	AF = 37, CE = 98.6%	[54]
SU-8	w = 1.3 μm g = 2.7 μm	2D C-IDEA	AF = 13, CE = 98%	[119]
SU-8, Pt	w < 2 μm, g < 3 μm	2D C-Pt IDEA	CE = 68%, 31% higher than C-C IDEA	[120]
SU-8 2005, SU-8 2075	(1) g = 5 µm, (2) 2D C-IDEA: h = 0.4 µm, w = 3.6 µm (3) 3D C-IDEA: h = 11 µm, diameter = 1.4 µm	3D carbon pillars on top of 2D C-IDEA	CV: 168 ± 12 mV for carbon 3D IDEA with pillars of 1.4 µm in diameter (aspect ratio of 8)	[129]
SU-8 2035, SU-8 2075	2Dp-25 and 3D#-25 bottom IDEA and pillars: w_bot =_ 25 µm, s_bot_ 25 µm, t_bot_ = 17 µm, d = 20 µm, s_pil_ = 60 µm, h_pil_ = 100 µm, 3D#-25 suspended IDEA: w_sus =_ 10 µm, s_sus_ = 20 µm, t_sus =_ 17 µm	2D C-IDEA and 3D C-IDEA with suspended	3D#-25: I_p_ = 0.527 ± 0.003 mA, 2D_p_ – 25: I_p_ = 0.23 ± 0.02 mA	[145]

AF = amplification factor, CE = collection efficiency.

**Table 3:** IDEA based-electrochemical sensor in biomedical applications using amperometric and impedimetric detections.

Techniques	Substrate/Electrode Materials	IDEA Dimensions	IDEA Structure	Sensor Performance	Ref.
Amperometric	Pt	w = g = h = 100 nm	Pt-nIDEA	AF = 161 for FcMeOH, CE = 99% -no biosensing test	[18]
Impedimetric	Polyimide sheet	w = 917 µm, g = 553 µm		2.5 CFU/mL of E. Coli detection	[102]
Impedimetric	Au	w = g = 10 μm, h = ∼60 nm	PPy/CNT film on 2 Au 2D IDEA	LOD: 28 ng/mL CysC	[105]
Amperometric and Impedimetric	Fe	(a) w = 20 μm, g = 15 μm, h = 80 μm, (b) w = 25 μm, g = 25 μm, h = 5 μm, (c) w = 25 μm, g = 25 μm, h = 20 μm, and (d) w = 25 μm, g = 25 μm, h = 80 μm.	3D VACNT IDEA	LOD: 1 ng/mL F-biotin	[136]
Impedimetric	SU-8 2002, SU-8 2025	Suspended carbon mesh: w ~300 nm, IDEA g ~1.9 µm	Suspended carbon mesh on top of the 2D C-IDEA	LOD: 0.43 pg/mL cMyo human serum	[139]
Amperometric, chronocoulometric	ITO	w = 5 μm, g = 10 μm between the bottom and ceiling and h = several tens µm	Closed 2D IDEA and 3D IDEA	LOD: 10 fg/mL (3D IDEA) and ∼100 fg/mL (Closed-2D IDEA) for mouseIgG 3D IDEA: 100 fg/mL for cTnI	[141]
Amperometric, Impedimetric	ITO electrode modified with PMG and PDA	w = 5 μm, g = 10 μm, h = 30 μm	3D IDEA without reference and counter electrodes.	LOD: 0.32 pg/mL of Creatine Kinase-MB	[146]
Amperometric and Impedimetric	Fe	w = g = 25 µm, h = 75 µm	3D VACNT IDEA	LOD: 0.24 pg/mL of CIP2A in salivasupernatant	[151]
Amperometric	SU-8 2002	w = 620 nm, h = 650 nm and g = 1.9 µm	AuNPs on top of 3D C-IDEA	LOD: ~1.28 µM of cholesterol	[168]
Impedimetric	Cr/Au	w = 10 µm, g = 100 µm	2D IDEA with porous sensor on top	LOD: 10 ng/mL for an IgG	[180]
Conductometric	Au	w = g = 20 μm	2D IDEA	LOD: 15 µM of ATP	[183]

**Table 4:** Integration of IDEA with nanomaterials and various source materials for wide ranging applications between years 2021 and 2022.

IDEA Source Materials	Integration in IDEA	Applications	Sensor Performance	Ref.
Ti/Pt IDEA	CuO–ZnO radial core–shell heterojunction nanowire arrays on metallic IDEA	photodetectors	responsivity: 26.3 A/W, detectivity: 5.8 × 1013 Jones	[184]
Metal IDEA on PET substrates	grown zinc oxide nanorod (NR) arrays cross-linked with IDEA	bending detection characteristics and sensing mechanism	no plasma treatment: highest gauge factor of 196 at a bending strain of 1.75% in the convex direction	[185]
C-Pt-IDEA	TiO_2_ nanoparticles	photoelectrochemical (PEC) water splitting	shining of 365 nm LED light	[186]
Ti/Pt IDEA onglass substrates	one IDEA activated by enzyme immobilization with HRP	capacitive detection of the H_2_O_2_ vapor/aerosol	sensitivity of 57.8 nF/c(H_2_O_2_), the response time (<60 s)	[187]
TaSi_2_ 3D-IDEA	with 4 µm high insulating barriers	detection ofcyanobacteria cells	LOD: 100 cells·mL^−1^	[188]
Au-IDEA	carbon nanodiamond	detection of SARS-CoV-2 nucleocapsid protein (NCP)	LOD: 0.389 fM	[189]
IDEA on polycarbonate substrates to make printed capacitive sensors	Ag nanoparticles	automotive infotainment	capacitance is increased when thickness increases	[190]
IDEA on ITO glass	carbon aerogel (CA)-polyaniline (PANI) composites	H_2_S gas sensing	PANI-CA-3 sensitivity:452%	[191]
3D IDEA micro-supercapacitors (MSCs)	Si/C/CNT@TiC composite nanostructure	alternating current line filtering	capacitance: 7.42 mF cm^−2^ (3.71 F g^−1^) at 5 mV s^−1^	[192]
IDEA capacitor on woven fabric	-	tactile sensor	capacitance change- 1.28 pF/gm.	[193]

## Data Availability

Not applicable.
